# Fetal growth and body size genes and risk of childhood acute lymphoblastic leukemia

**DOI:** 10.1007/s10552-012-0035-6

**Published:** 2012-07-28

**Authors:** Anand P. Chokkalingam, Catherine Metayer, Ghislaine Scelo, Jeffrey S. Chang, Joshua Schiffman, Kevin Y. Urayama, Xiaomei Ma, Helen M. Hansen, James H. Feusner, Lisa F. Barcellos, John K. Wiencke, Joseph L. Wiemels, Patricia A. Buffler

**Affiliations:** 1 School of Public Health, University of California Berkeley, Berkeley, CA USA; 2 International Agency for Research on Cancer, Lyon, France; 3 National Institute of Cancer Research, National Health Research Institutes, Tainan, Taiwan ROC; 4 Huntsman Cancer Institute, University of Utah, Salt Lake City, UT USA; 5 Yale University School of Public Health, New Haven, CT USA; 6 Department of Neurological Surgery, University of California San Francisco, San Francisco, CA USA; 7 Children’s Hospital and Research Center Oakland, Oakland, CA USA; 8 Department of Epidemiology and Biostatistics, University of California San Francisco, San Francisco, CA USA; 9 Division of Epidemiology, UC Berkeley School of Public Health, 1995 University Ave, Ste 460, Berkeley, CA 94704 USA

**Keywords:** IGF, Insulin-like growth factors, Leukemia, Childhood cancer, Fetal growth

## Abstract

Accumulating evidence suggests that childhood acute lymphoblastic leukemia (ALL) may be initiated in utero or early in the postnatal period. High birth weight (or rapid fetal growth) is associated with risk of ALL, but the mechanisms are not understood. In a population-based epidemiologic study of childhood ALL, we utilized a haplotype-based approach to assess the role of eight genes involved in fetal growth and body size regulation in 377 childhood ALL cases and 448 controls. We found significant haplotype associations with risk of childhood ALL for *IGF1* among non-Hispanics and Hispanics together (*p* = 0.002), for *IGF2* among Hispanics (*p* = 0.040), and for *IGF2R* among Hispanics and non-Hispanics (*p* = 0.051 and 0.009, respectively). No haplotype associations were observed for *IGF1R* or the studied genes involved in body size regulation, including *LEP*, *LEPR*, *GHRL*, and *NPY*. Our study is the first to identify an association between the genes involved in the IGF axis and risk of childhood ALL. These findings for childhood ALL emphasize the importance of fetal growth, when lymphoid progenitor cells are not yet fully differentiated and therefore more susceptible to malignant transformation. Additional studies are needed to confirm these findings and identify specific causal variants.

## Introduction

Leukemia is the most common cancer among children under 15 years of age, accounting for 32 % of all childhood malignancies [1, 2]. Acute lymphoblastic leukemia (ALL) is the most common subtype of childhood leukemia, comprising ~80 % of total disease [1]. The etiology of most cases of ALL in children is unclear, with few identified risk factors to date. A recent meta-analysis of epidemiological studies found that high birth weight (≥4,000 g) was associated with a modest but significantly elevated risk of childhood ALL (OR = 1.23; 95 % CI, 1.15–1.32) [3]. Other studies have probed this association with greater sophistication, taking into account gestational age, maternal height, and other factors to estimate the expected birth weight; the results show that higher than expected birth weight is associated with increased risk of childhood ALL, even among children who do not have high birth weight [4, 5]. These findings suggest that accelerated fetal growth, rather than high birth weight itself, is associated with childhood ALL risk, pointing to a potential etiologic role for factors regulating growth during early life.

Among the most important regulators of fetal growth are the components of the insulin-like growth factor (IGF) axis, including the growth factors IGF1 and IGF2, receptors IGF1R and IGF2R, and a number of binding proteins. While both IGF1 and IGF2 influence growth in utero, IGF1 continues to affect growth postnatally as well [6]. Circulating levels of IGF1 have been positively correlated with birth weight, birth length, and ponderal index (a measure of body leanness) [6]. Regulators of energy intake and expenditure, which play a role in body size and growth throughout life, may also have a key role in leukemogenesis through modulation of growth during the perinatal period [7]. Specifically, neuropeptide Y and ghrelin stimulate hunger, while the adipose-derived hormone leptin signals satiation. Importantly, variants in genes encoding members of the IGF axis and body size regulation pathway have been linked to birth weight [8–10].

In order to investigate the role of genes regulating fetal growth in childhood ALL risk, we utilized a haplotype-tagging approach to characterize variation in fetal growth and body size regulation genes in a population-based study of 377 ALL case children and 448 control children in Northern and Central California. To our knowledge, no previous studies have examined the role of fetal growth or body size regulation genes in the etiology of childhood ALL.

## Materials and methods

### Study population

The study was conducted among children participating in the Northern California Childhood Leukemia Study (NCCLS), a population-based case–control study conducted since 1995. The study enrollment and recruitment procedures have been described in detail previously [11]. Briefly, case children with incident childhood leukemia were ascertained via a rapid reporting system between the study office and participating hospitals. Control children were randomly selected from the California birth records and matched to case children on date of birth, sex, race, and Hispanic ethnicity (a child was considered Hispanic if one or both parents reported Hispanic ethnicity). Data on demographic factors and potential risk factors were elicited in person from a biological parent (usually the mother) by trained interviewers using a structured questionnaire.

This study was reviewed and approved by institutional review committees at the University of California Berkeley, the California Department of Public Health, and the participating hospitals. Written informed consent was obtained from all parent respondents.

### DNA specimens

Buccal specimens were collected with cytobrushes from participating children at interview and processed by heating in the presence of 0.5 N NaOH. Isolated DNA was later re-purified using an automated DNA extraction system (AutoGen, Holliston, MA, USA) and whole-genome amplified using GenomePlex reagents (Rubicon Genomics, Ann Arbor, MI, USA). When buccal cytobrush DNA was inadequate (26.6 % of subjects), DNA was isolated from dried bloodspots (DBS) collected at birth and archived by the Genetic Diseases Screening Program of the California Department of Public Health. After extraction (QIAamp 96 DNA Blood Kit, QIAGEN, Germany), these DNA samples were amplified using REPLI-g reagents (QIAGEN). All DNA specimens were quantitated using human-specific Alu-PCR [12] to confirm a minimum level of amplifiable human DNA. Concordance of all genotypes in DNA samples extracted from paired buccal cell and DBS specimens from 9 subjects was 98.9 %.

### Genotyping

We focused on genes in the IGF axis, including *IGF1*, *IGF2*, *IGF1R*, and *IGF2R*, as well as other genes involved in modulation of body size, including leptin (*LEP*), leptin receptor (*LEPR*), ghrelin (*GHRL*), and neuropeptide Y (*NPY*). Using HaploView software [13] in conjunction with single nucleotide polymorphism (SNP) data from the 30 Caucasian trios in the HapMap project (Release 19, Build 34, www.hapmap.org) and the 23 Hispanics in the SNP500Cancer project (www.snp500cancer.nci.nih.gov), we applied the method of Gabriel et al. [14] to select haplotype-tagging SNPs (htSNPs) that captured at least 80 % of the haplotype diversity for common haplotypes (>5 % frequency) in either the Caucasian or Hispanic populations. Because Hispanics are a recently admixed ethnic group, and 42 % of our study population is Hispanic (at least one parent reporting Hispanic ethnicity), we placed special emphasis on capturing haplotype structures in Hispanics. To maximize capture of potential regulatory regions, we included 10 kb stretches both up- and down-stream from the gene boundaries reported in the UCSC Genome Browser. Finally, a set of ancestry informative markers (AIMs) was included for genotyping; these have been previously identified to distinguish Amerindian, African, and European populations [15], three populations that make up the genetic ancestry of Hispanics.

The selected htSNPs were genotyped in 385 ALL cases and 456 controls using a custom Illumina GoldenGate panel (Illumina, San Diego, CA, USA). We applied a GenCall threshold of 0.25, and htSNP-wise and subject-wise call rate thresholds of ≥90 % and ≥95 %, respectively. Genotypes for duplicate DNA specimens from 59 subjects showed 99.1 % concordance. HtSNPs with a minor allele frequency <5 % (*n* = 6) or with significant deviation from Hardy–Weinberg equilibrium in both Hispanic controls and non-Hispanic controls (*p* < 0.01 in both, *n* = 1) were excluded. After applying these data quality exclusions, data for 108 htSNPs in the 8 genes were available for 377 ALL cases and 448 controls. A total of 80 AIMs were successfully typed in these subjects.

### Statistical analysis

We used haplotype trend regression methods to estimate odds ratios (ORs) and 95 % confidence intervals (CIs) of childhood ALL associated with genetic variants after adjustment for the matching factors: age, sex, race, and Hispanic ethnicity. Using the set of AIMs, we have previously calculated individual estimates of Amerindian, African, and European genetic ancestry and found no evidence of major confounding by estimated genetic ancestry (>10 %) over and above adjustment for self-reported race and ethnicity [16]. Thus, we used stratification or adjustment for the self-reported factors in our regression analyses.

As a preliminary step prior to haplotype analysis, we tested for potential interactions of individual htSNPs with Hispanic ethnicity on a gene-by-gene basis using unconditional logistic regression and the likelihood ratio test at the 0.05 significance level, after adjusting for age, sex, and child’s race.

In haplotype analyses, we used a sliding window approach, as implemented in the haplo.stats package for R [17], which allows for adjustment for matching factors as well as other potential covariates, including birth weight. This approach examines sub-haplotypes using the full set of htSNP data for a given gene, with differently sized “windows” of adjacent alleles. This is an effective means of combining multi-locus data for Hispanics and non-Hispanics, as it is agnostic to potential ethnic differences in haplotype structure. Accordingly, if none of the individual htSNPs in a given gene interacted significantly with Hispanic ethnicity, data for Hispanics and non-Hispanics were combined for sliding window analyses of that gene. Otherwise, the sliding window analysis for that gene was conducted separately for Hispanics and non-Hispanics. Rare haplotypes (≤5 % frequency among controls) were combined together for risk estimation. We utilized GrASP [18] to display and visualize sliding window results, and haplotype trend regression [19] to estimate the magnitude of effect associated with risk haplotypes of the windows with the smallest global *p* values.

## Results

**Fig. 1:**
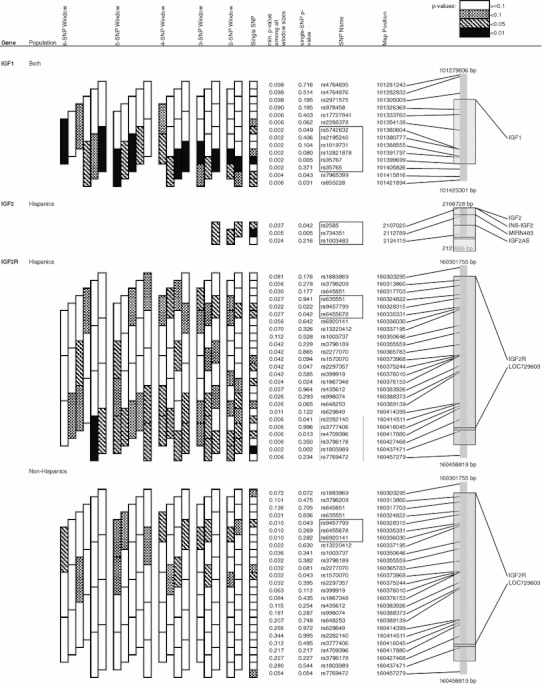
Significant (*p* ≤ 0.05) haplotype sliding window results for fetal growth and body size regulation genes and childhood ALL. Outlined blocks show a 6-SNP haplotype association for *IGF1* (*p* = 0.002), a 3-SNP haplotype association for *IGF2* (*p* = 0.037), and different but overlapping 3-SNP haplotype associations for *IGF2R* in Hispanics and non-Hispanics. The 6-SNP haplotype association observed for *IGF2R* among non-Hispanics was driven by rare haplotypes among controls (<5 % frequency) and therefore not considered further

Characteristics of the 377 ALL cases and 448 controls are shown in Table 1. As expected due to the matched case–control design of the NCCLS, the distribution of age, gender, race, and ethnicity was comparable. We examined a total of 108 htSNPs in 8 genes: *IGF1*, *IGF2*, *IGF1R*, *IGF2R*, *GHRL*, *LEP*, *LEPR*, and *NPY*. We found significant heterogeneity in the effects of htSNPs in 3 genes (*IGF2*, *IGF1R*, and *IGF2R*) between Hispanics and non-Hispanics. Haplotype sliding window analyses for these 3 genes were stratified by Hispanic ethnicity to permit detection of effects that might be apparent only by ethnicity, while those for the other 5 studied genes were conducted with both ethnic groups combined. Results for genes with significant (*p* ≤ 0.05) haplotype effects that persisted through increasingly larger windows are presented in Fig. 1. Haplotype trend regression results estimating the magnitudes of effect for haplotypes with the lowest multi-htSNP *p* value in sliding window analyses are shown in Table 2.

**Table 1:** Characteristics of genotyped childhood ALL cases and controls, NCCLS

Variable	Cases		Controls	
	*n*	%	*n*	%
Total	377	100.0	448	100.0
Sex				
Male	200	53.1	237	52.9
Female	177	46.9	211	47.1
Age at diagnosis or reference (year)				
Under 1	12	3.2	19	4.2
1–5	243	64.5	282	62.9
6–10	85	22.5	95	21.2
11–14	37	9.8	52	11.6
Ethnicity				
Hispanic	221	58.6	269	60.0
Non-Hispanic	156	41.4	179	40.0
Race				
White	214	56.8	255	56.9
Black	15	4.0	16	3.6
Native American	7	1.9	8	1.8
Asian/Pacific Islander	25	6.6	35	7.8
Mixed	110	29.2	133	29.7
Don’t know	6	1.6	1	0.2

**Table 2:** Haplotype trend regression results: fetal growth and body size regulation genes in childhood ALL risk, NCCLS

Gene	SNPs	Ethnic group	Haplotype	Control frequency^a^	Case frequency^a^	OR (95 % CI)	*p* value^b^	Global *p* ^c^
*IGF1*	(*rs5742632, rs2195240, rs1019731, rs12821878, rs35767, rs35765*)	Both	Rare haplotypes^d^	0.016	0.036	9.90 (2.07, 47.26)	**0.004**	**0.002**
			A-A-C-A-G-C	0.058	0.058	1.23 (0.51, 3.00)	0.644	
			G-G-C-G-A-C	0.076	0.106	2.13 (1.04, 4.35)	**0.039**	
			A-A-C-G-A-A	0.091	0.099	1.45 (0.72, 2.92)	0.304	
			A-A-A-A-G-C	0.138	0.168	1.81 (1.02, 3.22)	**0.044**	
			G-G-C-G-G-C	0.200	0.153	0.72 (0.41, 1.28)	0.268	
			A-A-C-G-G-C	0.420	0.379	1.00 (ref)		
*IGF2*	*(rs2585, rs734351, rs1003483)*	Hisp	Rare haplotypes^d^	0.024	0.049	9.34 (1.09, 80.14)	**0.042**	**0.040**
			G-G-C	0.053	0.080	2.92 (0.72, 11.90)	0.134	
			A-G-A	0.148	0.194	2.34 (1.00, 5.48)	**0.051**	
			G-A-A	0.226	0.216	1.30 (0.57, 2.98)	0.538	
			G-A-C	0.549	0.461	1.00 (ref)		
*IGF2R*	*(rs635551, rs9457799, rs6455678)*	Hisp	G-C-A	0.086	0.087	1.25 (0.41–3.80)	0.697	**0.051**
			A-A-A	0.095	0.048	0.29 (0.05–1.79)	0.184	
			A-C-G	0.271	0.340	2.10 (0.98–4.53)	**0.058**	
			A-C-A	0.549	0.525	1.00 (ref)		
*IGF2R*	*(rs9457799, rs6455678, rs6920141)*	NH	Rare haplotypes^d^	0.013	0.039	9.69 (1.64, 57.34)	**0.012**	**0.009**
			A-A-G	0.095	0.133	2.34 (1.02, 5.36)	**0.044**	
			C-G-G	0.201	0.172	0.82 (0.44, 1.52)	0.520	
			C-A-A	0.691	0.656	1.00 (ref)		

^a^377 childhood ALL cases, 448 controls

^b^Wald test; bold type indicates significant results

^c^Global test for haplotype association; bold type indicates significant results

^d^Includes any haplotypes with less than 5 % frequency among controls

Among all subjects, *IGF1* showed significant haplotype associations that persisted through progressively larger htSNP windows (Fig. 1). The selected six-htSNP haplotype window showed a stronger association (global *p* = 0.002) than any individual SNP (Table 2). In this window, haplotypes G-G-C-G-A-C and A-A-A-A-G-C were associated with significantly increased risk (OR = 2.13 and *p* = 0.039, and OR = 1.81 and *p* = 0.044, respectively).

The 3-htSNPs genotyped in *IGF2* showed a significant haplotype association among Hispanics, and the A-G-A haplotype was significantly associated with an increased risk of ALL (OR = 2.34, *p* = 0.051).

*IGF2R* showed significant haplotype associations in both Hispanics and non-Hispanics, but the haplotype windows showing the strongest associations differed between ethnic groups. Among Hispanics, the minimum *p* value among all haplotype windows was for a 6-htSNP window whose effects were driven by haplotypes with less than 5 % frequency among controls; accordingly, this 6-htSNP window was not considered further. However, there was also a 3-htSNP window in *IGF2R* that was significantly associated with childhood ALL risk among Hispanics (global *p* value = 0.051). The A-C-G haplotype of this window showed a borderline significantly increased risk (OR = 2.10, *p* = 0.058). Among non-Hispanics, a 3-htSNP haplotype window in which 2 htSNPs overlapped with the window observed for Hispanics was significantly associated with childhood ALL (global *p* = 0.009), and haplotype A-A-G was associated with a significantly increased risk (OR = 2.34, *p* = 0.044).

Of the htSNPs genotyped, four had putative function (non-synonymous variants, splice variants, variants in regulatory regions, or variants in strong linkage disequilibrium with functional variants) as listed in Entrez Gene. Risk estimates for these individual variants are shown in Table 3. Only one of these putative functional variants, rs35767 in *IGF1*, was found to have a significant association with risk of childhood ALL. Its minor allele was associated with a significantly increased risk of disease in both ethnicities combined (heterozygote: OR = 1.48; 95 % CI, 1.10–1.99; homozygote: OR = 1.74; 95 % CI, 0.88–3.45; *p*_trend_ = 0.005).

**Table 3:** Childhood ALL risk estimates for putative functional SNPs in fetal growth and body size regulation genes, NCCLS

Gene	SNP	Both ethnicities						
			*n* case	*n* ctrl	OR (95 % CI)	*p* ^a^	*p*_log add_^b^	*p*_int_^c^
*IGF1*	rs35767	(LD with functional microsatellite)						
		GG	217	300	1.00 (ref)		**0.005**	0.3190
		AG	140	131	1.48 (1.1, 1.99)	**0.010**		
		AA	20	16	1.74 (0.88, 3.45)	0.111		
*IGF2R*	rs629849	(G1619R)						
		GG	308	356	1.00 (ref)		0.376	0.1920
		AG	63	78	0.94 (0.65, 1.35)	0.720		
		AA	1	5	0.23 (0.03, 1.97)	0.179		
*HRL*	rs4684677	(Q78L)						
		TT	273	333	1.00 (ref)		0.231	0.8245
		AT	83	99	1.03 (0.72, 1.47)	0.880		
		AA	17	10	2.11 (0.93, 4.79)	0.074		
*GHRL*	rs696217	(L60M)						
		CC	322	370	1.00 (ref)		0.325	0.5753
		AC	49	68	0.83 (0.56, 1.24)	0.365		
		AA	1	2	0.59 (0.05, 6.58)	0.669		

^a^Wald test *p* value; bold type indicates significant results

^b^Log additive inheritance model; bold type indicates significant results

^c^Likelihood ratio test *p* value for interaction with Hispanic status

## Discussion

In this population-based case–control study, we examined the risk of childhood ALL associated with several genes involved in fetal growth and body size regulation, utilizing a haplotype-tagging approach to maximize capture of genetic variation. We identified haplotypes of several genes that were significantly associated with childhood ALL, including *IGF1*, *IGF2*, and *IGF2R*. To our knowledge, no previous studies have expressly examined the role of genes involved in the IGF axis or body size regulation in risk of childhood ALL. Our findings support the hypothesis that factors involved in fetal growth and body size regulation, specifically components of the IGF axis, play a role in mediating risk of childhood ALL.

In this study, we found two haplotypes in the same 6-htSNP window of *IGF1* to be significantly associated with an increased risk of childhood ALL. The haplotype window of association here stretches across 25 kb, from the 5′ promoter region into the second intron. IGF1, also known as somatomedin C, is expressed by both the mother and the fetus during pregnancy and plays a critical role in modulating cellular proliferation, differentiation, and apoptosis [6]. While IGF1 controls growth directly in utero, it becomes subject to regulation by growth hormone in the postnatal period [9].

We identified a significant 3-htSNP haplotype of *IGF2* associated with a markedly increased risk of childhood ALL. In contrast to *IGF1*, *IGF2* is thought to play a more critical role in growth and development in utero versus postnatally [6]. Serological IGF2 levels are higher during the in utero versus the postnatal period, and studies of *IGF2* knockout mice show stunted fetal growth but normal postnatal growth [20]. Although IGF2 is an imprinted gene, meaning that only one allele is usually expressed, there are no consistent findings to support the hypothesis that loss of imprinting of *IGF2* is associated with an increased risk of childhood ALL [6].

Finally, we found significant associations of overlapping but distinct *IGF2R* haplotype windows with risk of childhood ALL among Hispanics and non-Hispanics separately. Interestingly, the two overlapping htSNPs in each of these windows, rs9457799 and rs6455678, had significantly different single-htSNP effects in Hispanics versus non-Hispanics. The differential effect of these two htSNPs was present in the haplotype analysis as well: The risk haplotype among Hispanics included C-G at these two loci and was associated with a borderline significant increased risk, while among non-Hispanics the haplotype including C-G at these two loci showed no association. Conversely, the haplotype with A-A at these two loci was associated with a significantly increased risk among non-Hispanics and null risk among Hispanics. The *IGF2R* gene product serves no signal transduction purpose; rather, by binding IGF2, it appears to serve mainly to regulate IGF2 levels in utero [21, 22].

The putative functional *IGF1* SNP (rs35767) found to be significantly associated with childhood ALL risk is in the *IGF1* gene promoter region. It is not functional itself but is in strong linkage disequilibrium with -841(CA)_n_, a common microsatellite that has been linked to circulating IGF1 levels as well as birth weight and other measures of body size [10]. This SNP is part of the 6-htSNP haplotype window we found to be significantly associated with childhood ALL risk.

We also examined body size regulation genes outside the IGF axis. These genes include *LEP*, whose gene product leptin signals satiety and has been found to correlate with neonatal birth weight [23] and size for gestational age [24], and LEPR, whose gene product mediates the effects of leptin. Ghrelin, encoded by *GHRL* and secreted primarily by the stomach, serves to stimulate appetite and promote adiposity and may have a compensatory effect on intra-uterine growth restriction, serving to boost growth in the postnatal period [7]. Neuropeptide Y, encoded by *NPY*, is a neurotransmitter that also leads to increased appetite and storage of energy as fat, as well as decreased physical activity. However, we found no significant haplotype associations between these genes and risk of childhood ALL.

In this study, statistical adjustment for birth weight did not affect the associations observed for the significant risk haplotypes in *IGF1*, *IGF2*, and *IGF2R*, or the single nominally significant (*p* < 0.05) putative functional SNP in *IGF1*. The independence of the observed associations from birth weight, coupled with the absence of direct associations of the haplotypes and htSNP in question with birth weight (data not shown), suggests that the effects of the risk haplotypes and alleles on childhood ALL risk are not directly mediated by birth weight. Indeed, data from a recent meta-analysis of genome-wide studies did not identify birth weight–associated loci in IGF axis genes [25]. Because we did not begin collecting data on maternal height or pre-pregnancy weight until partway through recruitment of the study population, these data were unavailable for 49 % of study subjects, and therefore, we were unable to estimate percent optimal birth weight, a measure of appropriateness of fetal growth that takes into account maternal body size, gestational age, and other factors and may be more relevant to ALL risk than birth weight alone [4].

Maternal components of the IGF axis are unable to cross the placenta [26]; accordingly, fetal levels of these factors are likely to be determined by fetal tissues, lending support to the notion that fetal genotypes are particularly relevant. However, maternal genotypes influencing maternal metabolism and growth may have an impact on fetal growth; accordingly, investigation of maternal genotypes is warranted.

One of the key strengths of our study is the inclusion of U.S. Hispanics, an understudied population with the highest reported rates of childhood leukemia in California [27]. Our htSNP selection strategy included elements designed to maximize capture of genetic variation in Hispanics. We examined Hispanics separately from non-Hispanics where there was significant heterogeneity in between-group effects of individual htSNPs in each gene. Although this approach may have limited our ability to detect associations in the population as a whole, we felt it was necessary given that genetic susceptibility may be different in Hispanics versus non-Hispanics due to the Hispanic population’s relatively recent genetic admixture [15]. Results that differ between Hispanics and non-Hispanics may be due to differences in allele frequency and/or haplotype structure or may reflect underlying differences in exposures that modulate the effects of genes. Regardless, if the results are not spurious, they represent potential risk loci, and we present them in either or both ethnic groups for replication and further follow-up. The limited size of racial/ethnic sub-populations within the non-Hispanic group precluded further stratification of this group; as such, genetic heterogeneity within this group might have obscured results. However, adjustment for calculated genetic ancestry did not markedly change the race- and ethnicity-adjusted results we present here, indicating that the potential impact of population stratification is minimal.

Two large genome-wide association studies on childhood ALL have been published to date (with 907 cases and 2,398 adult and child controls, and 317 cases and 17,958 adult controls, respectively) [28, 29]. These studies have identified a number of novel loci; however, no significant associations were observed for genes in IGF axis and body size regulation genes. This may be due to stringent multiple testing adjustment (at the *p* ≤ 1 × 10^−7^ level) to account for the large number of individual variants tested in genome-wide studies. In contrast to the agnostic approach to discovery used in such studies, our study focused on relatively few genes representing key elements of the IGF axis and body size regulation pathways. It commenced prior to the recent publication of results linking birth weight to loci in two genes (*ADCY5* and *CCNL1*) [25]; as such, we were unable to investigate these genes. We concede that our study results may be due to chance and therefore must be replicated. However, the haplotype-tagging approach we adopted maximizes capture of total variation within each candidate gene, and the haplotype analysis increases statistical power to detect associations over analyses of individual variants. Finally, although the haplotype-tagging approach does not pinpoint potential causal variants, it does localize risk-associated regions for additional study, including fine-mapping.

Recent studies showing correlations of umbilical cord serum IGF1 levels with both cord stem cell levels and birth weight [30, 31] support the notion that increased fetal growth may increase cancer risk by either increasing pre-leukemic stem cell populations targeted for initiation, or promoting growth or survival of an initiated leukemic stem cell. Together with this, our results are consistent with the notion that the IGF axis influences the initiation or promotion of childhood ALL early in life. Further studies are needed to determine the specific roles played.

In summary, we set out to investigate the role of genes in the IGF axis and body size regulation pathways in risk of childhood ALL. Our results indicate that elements of the IGF axis are associated with childhood ALL risk. These findings are consistent with the evidence that childhood ALL initiation and/or promotion may begin in utero, when lymphoid progenitor cells are not yet fully differentiated and therefore more susceptible to malignant transformation. The associations and interactions identified here should be considered targets for replication in additional studies with larger sample size and finer coverage of variants in the identified associated gene regions.
